# Low Maternal DLK1 Levels at 26 Weeks Is Associated With Small for Gestational Age at Birth

**DOI:** 10.3389/fendo.2022.836731

**Published:** 2022-02-28

**Authors:** Aurelie Pham, Delphine Mitanchez, Anne Forhan, Laurence Perin, Yves Le Bouc, Frederic Brioude, Marie-Laure Sobrier, Barbara Heude, Irene Netchine

**Affiliations:** ^1^ Sorbonne Université, INSERM, Centre de Recherche Saint Antoine, APHP, Hôpital Armand Trousseau, Service de Néonatologie, Paris, France; ^2^ Sorbonne Université, INSERM, Centre de Recherche Saint Antoine, Paris, France; ^3^ Centre Hospitalier Régional Universitaire (CHRU) de Tours, Hôpital Bretonneau, Service de Néonatologie, Tours, France; ^4^ Université de Paris Cité, INSERM, INRAE, Centre of Research in Epidemiology and StatisticS (CRESS), Paris, France; ^5^ Sorbonne Université, APHP, Hôpital Armand Trousseau, Explorations Fonctionnelles Endocriniennes, Endocrinologie Moléculaire et Pathologies d’Empreinte, Paris, France; ^6^ Sorbonne Université, INSERM, Centre de Recherche Saint Antoine, APHP, Hôpital Armand Trousseau, Explorations Fonctionnelles Endocriniennes, Endocrinologie Moléculaire et Pathologies d'Empreinte, Paris, France

**Keywords:** DLK1, small for gestational age (SGA), fetal growth restriction, placental vascular dysfunction, biomarker

## Abstract

Detecting SGA (small for gestational age) during pregnancy improves the fetal and neonatal prognosis. To date, there is no valid antenatal biomarker of SGA used in clinical practice. Maternal circulating DLK1 (delta-like non-canonical notch ligand 1) levels have been shown to be significantly lower in pregnant women at 36 weeks of gestation (WG) who delivered a SGA newborn than in controls. Data in the literature are contradictory on the association between maternal circulating DLK1 levels and placental vascular dysfunction. The objective was to determine if maternal DLK1 levels in the second trimester of pregnancy are predictive of SGA, and to assess whether the measurement of DLK1 levels in maternal blood could be a means to distinguish SGA with placental vascular dysfunction from that due to other causes. We conducted a nested cased-control study within the EDEN mother-child cohort. 193 SGA (birth weight < 10^th^ percentile) and 370 mother-child control (birth weight between the 25^th^ and 75^th^ percentile) matched pairs were identified in the EDEN cohort. Maternal circulating DLK1 levels at 26 WG were significantly lower for children born SGA than for controls (27.7 ± 8.7 ng/mL *vs* 30.4 ± 10.6 ng/mL, p = 0.001). Maternal blood DLK1 levels in the first quartile (DLK1 < 22.85 ng/mL) were associated with an odds ratio for SGA of 1.98 [1.15 - 3.37]. DLK1 was less predictive of SGA than ultrasound, with an area under the curve of 0.578. Maternal circulating DLK1 levels were not significantly different in cases of SGA with signs of placental vascular dysfunction (n = 63, 27.1 ± 9.2 ng/mL) than in those without placental dysfunction (n = 129, 28.0 ± 8.5 ng/mL, p = 0.53). The level of circulating DLK1 is reduced in the second trimester of pregnancy in cases of SGA at birth, independently of signs of placental vascular dysfunction. However, DLK1 alone cannot predict the risk of SGA.

## Introduction

Fetal growth restriction (FGR), defined as a failure of the fetus to reach its genetically determined growth potential (1), is one of the most common causes of perinatal mortality and morbidity. FGR often results in neonates who are small for gestational age (SGA), usually defined as a birth weight < 10^th^ percentile of a given reference by neonatologists or a birth weight and/or height of < -2 standard deviations of the reference mean by endocrinologists (2, 3).

The performance of ultrasound to detect SGA during pregnancy is low, with a sensitivity of 10 to 30% in the general population and a positive predictive value of 50% (4). However, detecting SGA during pregnancy in the second or third trimester of gestation has been shown to improve the fetal and neonatal prognosis and may be associated to long term metabolic consequences (5–7). A study including 92,218 singletons reported a lower stillbirth rate in antenatal-detected versus non-detected SGA (9.7 *vs*18.9 per 1000 births) (8). Another recent study including 2425 FGR children reported that stillbirth risk was increased in undetected FGR fetuses (OR 2.63) (9). FGR results from multiple causes (10). One of the main causes of SGA is placental vascular dysfunction. Placental vascular dysfunction, due to abnormal placentation with inadequate remodeling of maternal spiral and altered uteroplacental blood perfusion, impairs materno-fetal exchange of nutrients and oxygen leading to FGR (11–13). The identification of SGA with placental vascular dysfunction is clinically relevant because of poorer perinatal outcomes and higher risks of severe neonatal comorbidities than for SGA without placental vascular dysfunction were reported (10, 14, 15). Repeated estimations by ultrasound of fetal weight (EFW) and abdominal circumference, Doppler-flow velocimetry of umbilical artery (UA) and fetal middle cerebral artery (MCA) and pulsatility index in uterine artery (UtA) are currently used to diagnose FGR with placental vascular dysfunction (16). Indeed, a high mean pulsatility index in UtA reflects the lack of physiological transformation of the uterine arteries from high- to low-resistance vessels. This is associated with maternal vascular malperfusion of the placenta and consequently of the fetus, leading to fetal hypoxia (13). On the fetal side, Doppler velocimetry allows the evaluation of cerebroplacental ratio (ACM pulsatility index/UA pulsatility index) that reflects the cardiovascular adaptation of the fetus to hypoxia (13).

However, another promising approach is to combine ultrasonic data with maternal biomarkers of placental dysfunction (1). To date, no antenatal predictive biomarker of SGA are used in clinical practice (1, 14, 17).

DLK1 (delta-like non-canonical notch ligand 1) is the product of an imprinted gene that is expressed during fetal development (18). Parental imprinting is an epigenetic mechanism that refers to the monoallelic silencing of genes according to their parental origin. In the 14q32 imprinted region, the imprinting control center *MEG3/DLK1:IG-DMR* is methylated on the paternal allele (19), resulting in *DLK1* expression from the same allele. DLK1 is a single-pass transmembrane protein that is cleaved by extracellular proteases to give rise to a circulating form, which reaches a high concentration in the maternal circulation during pregnancy (20), as well as in the amniotic fluid and fetal circulation during the second and third trimesters (18). DLK1 plays an important role in preserving the pool of various progenitor cells until they differentiate (18) and has an impact on metabolism. *DLK1*, also called *PREF-1* (preadipocyte factor 1), is known for its involvement in adipogenesis (21, 22). *Dlk1* null mice display accelerated weight gain and hyperlipidemia (22) and patients with *DLK1* mutations show metabolic alterations in adulthood (23). Data show that DLK1 is present in the extra-embryonic tissue as early as five weeks of gestation (WG) and then until birth (18). Cleaton et al. showed maternal circulating DLK1 levels to be positively associated with embryonic mass during mouse gestation and to be required for maternal metabolic adaptations to pregnancy. According to Cleaton, DLK1 levels are significantly lower at 36 WG for pregnant women who later delivered a SGA newborn than controls. In the same study, maternal circulating DLK1 levels were also associated with placental vascular dysfunction and FGR (20). MacDonald et al. recently confirmed low circulating maternal DLK1 levels at 28 and 36 WG for women who finally delivered a SGA newborn, but they did not find any significant association with placental vascular dysfunction (24).

Evaluation of the association between maternal DLK1 levels and fetal growth at an earlier gestational age would offer a greater clinical benefit. We aimed to determine whether the level of DLK1 in maternal serum during the second trimester of gestation is a predictive biomarker of SGA and to assess whether the measurement of DLK1 levels in maternal blood could be a means to distinguish SGA with placental vascular dysfunction from that due to other causes.

## Materials and Methods

### The EDEN Mother-Child Cohort

This is a nested cased-control study based on the EDEN mother-child cohort (25) that was set up in 2003 in two university maternity clinics, Nancy and Poitiers, France. Participation in the EDEN mother-child cohort was proposed to all women visiting the prenatal clinic before 24 WG. Exclusion criteria were multiple pregnancies, known diabetes before pregnancy, the inability to read and understand French, or planning to move out of the region within the next three years. Among the 3,758 women invited to participate in the EDEN mother child-cohort, 2,002 (53%) were enrolled in the study (1,034 women from Nancy and 968 from Poitiers).

### Data Collection

Data were collected from medical and obstetrical records and clinical examinations. Mothers underwent a clinical examination with blood sampling at 24 to 28 WG. At birth, the weight of the newborns was measured by midwives using an electronic scale (Seca Ltd). Data on maternal smoking during pregnancy and maternal and newborn characteristics were collected during pregnancy or at birth. Maternal weight before pregnancy was obtained by interview at inclusion and maternal height measured during the clinical examination. Both anthropometric measurements were used to calculate the BMI as the reported weight (kilograms) divided by the square of the measured height (meters). Information on ultrasound measurements and placental function measured at 26 WG were collected from obstetrical records: fetal head circumference, fetal abdominal circumference, femur length, amniotic fluid index, notch in Doppler flow velocimetry of the uterine artery, pulsatility index, and reverse flow on umbilical doppler. Information on the presence of a maternal vascular disease before pregnancy (chronic high blood pressure, sickle cell anemia, antiphospholipid or anticardiolipin antibody syndrome, or autoimmune disease) was also collected from obstetrical records.

### Case-Control Study Design

We conducted a case-control study nested within the EDEN mother-child cohort. Cases were defined as mothers who gave birth to newborns with a birth weight < 10^th^ percentile according to customized fetal growth charts (26). For each case, two matched controls were recruited among mothers who gave birth to AGA (adapted for gestational age) newborns with a birth weight between the 25^th^ and 75^th^ percentile of the same customized references (26). Controls were matched for the maternity clinic (Nancy – Poitiers), sex of the child, maternal age, maternal body mass index (BMI) (4 groups: < 18.5 kg/m^2^, 18.5-24.9 kg/m^2^, 25-30 kg/m^2^, > 30 kg/m^2^), and gestational age (GA) at blood collection. In total, 196 SGA mother-child pairs and 370 match control pairs were identified in the EDEN mother-child cohort.

### Definition of SGA With Signs of Placental Vascular Dysfunction

SGA children were considered to be SGA with placental vascular dysfunction when they met at least one of the following criteria (1, 27): fetal head circumference > 10^th^ percentile and fetal abdominal circumference < 10^th^ percentile, presence of a notch in Doppler-flow velocimetry of the UtA, high-resistance UtA flow, defined as a mean pulsatility index > 95^th^ percentile, reverse flow on umbilical doppler, high-resistance UA flow, defined as a pulsatility index > 95^th^ percentile, or the presence of a maternal vascular disease before pregnancy (chronic high blood pressure, sickle cell anemia, antiphospholipid syndrome).

#### Quantitative Determination of DLK1 Levels in Serum

Serum samples were frozen on the day of collection and stored at -80°C. The DLK1 concentration was measured blind to the case/control status. Maternal serum DLK1 levels were quantified in 96-well plates using the DLK1 soluble (human) ELISA Kit (Adipogen Life sciences, Coger, Switzerland), based on the sandwich enzyme linked-immunosorbent assay (ELISA). Maternal serum samples were diluted 1/10 with buffer (Adipogen Life sciences, Coger, Switzerland) containing fetal bovine serum (0.5%) and Tween (0.05%) before binding to the antibody-coated plates. The human DLK1 ELISA kit was then used according to the manufacturers’ instructions.

#### Statistical Analysis

Characteristics of the mothers and children are described using means ± SD and frequencies (%). Differences between cases and controls were tested using Student *t* and chi-square tests for continuous and categorical variables, respectively. Circulating DLK1 levels were compared according to maternal and pregnancy characteristics using Student *t* tests or ANOVA for multiple means comparisons. Univariate analysis was performed by the analysis of variance for qualitative variables or the Pearson correlation coefficient for quantitative variables. The effect of placental vascular dysfunction was assessed using an interaction test. The association between circulating DLK1 levels and SGA was investigated using unconditional logistic multiple regression models, adjusted for matching factors: center, sex of the child, gestational age, maternal age, pre-pregnancy BMI, and gestational age at blood collection. Two models were computed, one with DLK1 considered as a continuous trait and the other with DLK1 considered as a categorical variable divided into quartiles.

We assessed the discriminative power of DLK1 alone or in combination with ultrasound measurements by examining the ROC curves of the respective models. The areas under the ROC curves (AUROCs) were measured using Mann-Whitney tests and compared using DeLong tests. This analysis was conducted using the data from 520 subjects (180 cases and 340 controls) due to missing values for the ultrasound data. We conducted sensitivity analyses to determine whether further adjustment for smoking during pregnancy modified the results. Statistical analyses were performed using SAS**^®^** 9.3 with AIX 7.1, with P < 0.05 considered to be statistically significant.

### Ethics Approval and Consent to Participate

The EDEN mother-child cohort received approval from the ethics committee of the Kremlin Bicêtre Hospital (France) on 12 December 2002 and from the French data privacy institution CNIL. All participants were treated in accordance with French national ethics regulations.

## Results

### DLK1 Levels at 26 WG Are Lower for Women Who Give Birth to SGA Infants

**Figure 1:**
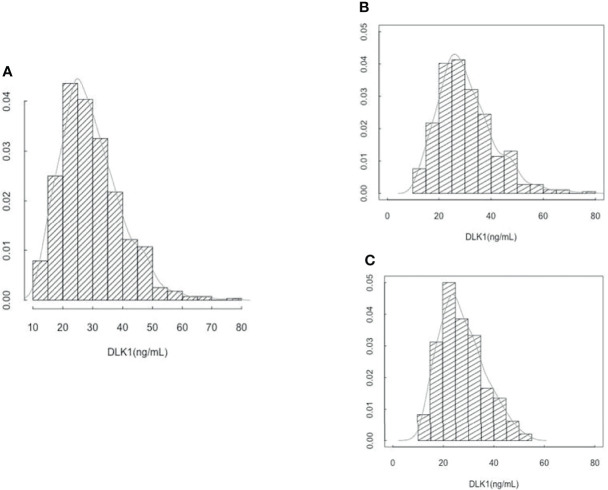
**(A)** Distribution of circulating DLK1 levels at 26 weeks of gestation in 560 EDEN mothers. **(B)** Distribution of circulating DLK1 levels at 26 weeks of gestation in 368 mothers who gave birth to AGA newborn. **(C)** Distribution of circulating DLK1 levels at 26 weeks of gestation in 192 mothers who gave birth to SGA newborn.

Among the 193 identified cases of SGA, the DLK1 value was aberrant in the maternal serum of one SGA case (870.7 ng/mL *vs* a mean value of, for the other 562 samples, 29.5 ± 10.1ng/mL). After exclusion of this subject and her matched controls, 192 SGA and 368 matched controls were included in the analyses. Sixty-three cases of SGA (32.6%) were classified in the subgroup of SGA with placental vascular dysfunction. The distribution of DLK1 values is presented in **Figure 1**.

Characteristics of the two groups are presented in **Table 1**. The median gestational age at blood sampling was 26 WG. As expected, there were no differences between cases and controls concerning the matching factors. The proportion of women who declared smoking during pregnancy was no different between cases and controls.

**Table 1:** Characteristics of the matched case and control pregnancies from the EDEN mother-child cohort.

n	All	Control	Case	p
	560	368	192	
**Medical Center**				0.93
Poitiers	41.4% (232)	41.3% (152)	41.7% (80)	
Nancy	58.6% (328)	58.7% (216)	58.3% (112)	
**Sex**				0.88
Female	46.4% (260)	46.2% (170)	46.9% (90)	
Male	53.6% (300)	53.8% (198)	53.1% (102)	
**Maternal age** (years)	29.3 ± 4.5	29.3 ± 4.5	29.3 ± 4.7	0.91
**BMI** (kg/m^2^)	23.1 ± 4.5	23.0 ± 4.4	23.3 ± 4.7	0.51
< 18.5	10% (56)	10.6% (39)	8.9% (17)	0.80
[18.5-25]	64.6% (362)	65.2% (240)	63.5% (122)	
[25-30]	17.7% (99)	16.5% (62)	19.3% (37)	
≥ 30	7.7% (43)	7.3% (27)	8.3% (16)	
**Gestational age at blood sampling** (WG)	26.2 ± 1.2	26.3 ± 1.2	26.2 ± 1.2	0.79
Smoking during pregnancy^*^				
No	71.2% (398)	73.3% (269)	67.2% (129)	0.13
Yes	28.8% (161)	26.7% (98)	32.8% (63)	

^*^Information was missing for one woman.

There were also no differences in DLK1 levels according to the center of inclusion or the sex of the neonates (**Table 2**). DLK1 levels in maternal blood serum at 26 WG were significantly lower for women who gave birth to SGA infants (27.7 ± 8.7 ng/mL) than for control women (30.4 ± 10.6 ng/mL, p = 0.001) (**Table 2**).

**Table 2:** DLK1 maternal serum levels at 26 weeks of gestation according to maternal and neonatal characteristics.

Cases/Controls	n	m ± sd	p
			
Controls	368	30.4 ± 10.6	< 10^-3^
Cases (SGA)	192	27.7 ± 8.7	
**Medical Center**			0.75
Poitiers	232	29.3 ± 10.6	
Nancy	328	29.6 ± 9.9	
**Sex**			0.36
Male	300	29.1 ± 9.4	
Female	260	29.9 ± 10.7	
**Placental vascular dysfunction**			0.009
Controls	368	30.4 ± 10.6	
Cases (SGA) without signs of placental vascular dysfunction	129	28.0 ± 8.5	
Cases (SGA) with signs of placental vascular dysfunction	63	27.1 ± 9.2	
**Smoking during pregnancy**			
No	398	29.9 ± 10.5	0.07
Yes	161	28.4 ± 8.8	

Consideration of DLK1 as continuous variable in the multiple regression analysis showed a significant association of DLK1 levels with SGA: a 1ng/mL increase in DLK1 decreased the risk of SGA by 3%. Consideration of DLK1 as a categorical variable divided into quartiles showed an odds ratio for SGA of 1.98 [1.15 - 3.37] in the first quartile compared to the last (**Table 3**).

**Table 3:** Multivariable logistic regression analyses of DLK1 as a predictor of risk of SGA (N = 560).

	Odds Ratio [95%IC]	p-value
DLK1 (for 1 ng/mL)	0.97 [0.95 - 0.99]	0.003
Separately according to center		
Poitiers (n = 232)	0.97 [0.940 - 1.000]	0.052
Nancy (n = 328)	0.97 [0.946 - 0.996]	0.025
DLK1 Quartiles (ng/mL)		0.013
Q1 < 22.85	1.98 [1.15 - 3.37]	
Q2 22.85-28.05	1.75 [1.03 - 2.96]	
Q3 28.05-35.20	1.52 [0.90 - 2.54]	
Q4 > 35.2	ref	

Models consist of unconditional logistic regressions adjusted for matching variables: center, sex of the child, maternal age, gestational age at sampling, maternal BMI. Ref, reference.

### DLK1 Levels Are Lower for Women Who Give Birth to SGA Infants Independently of Placental Vascular Dysfunction

Maternal circulating DLK1 levels were not significantly different in cases of SGA with signs of placental vascular dysfunction (n = 63, 27.1 ± 9.2 ng/mL) than in those without placental dysfunction (n = 129, 28.0 ± 8.5 ng/mL, p = 0.53). Indeed, they both showed significantly lower levels of maternal circulating DLK1 than controls (n = 368) (30.4 ± 10.6 ng/mL, p = 0.009) (**Table 2**).

### DLK1: A Predictive Biomarker of SGA?

**Figure 2:**
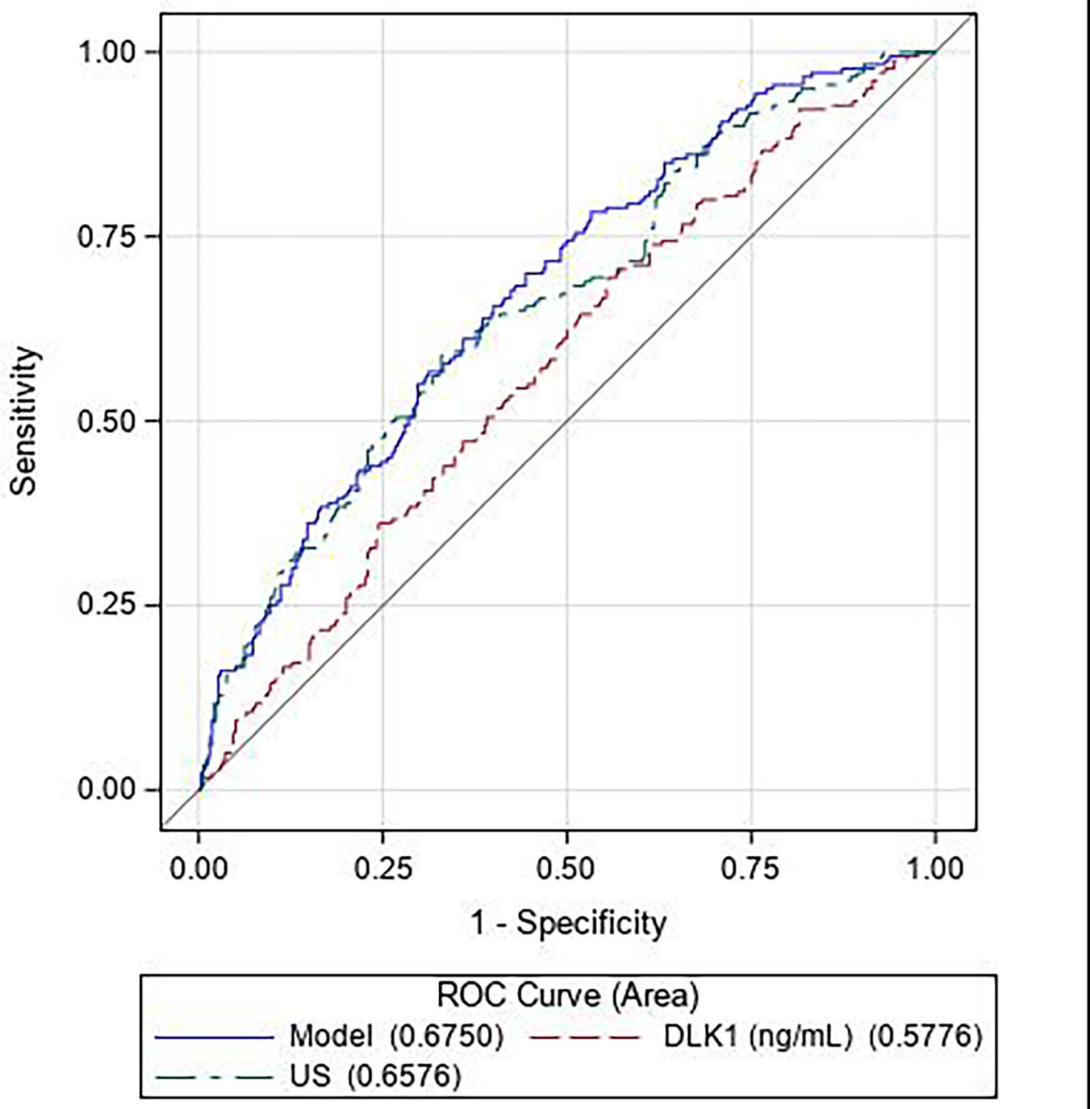
Receiver operating characteristic (ROC) curves for the predictive performance of DLK1 alone (*— — —*, red), ultrasound data (fetal head circumference, fetal abdominal circumference, femur length, and gestational age at measurement) alone (*— - —*, green), and DLK1 and ultrasound data combined (*——−*, blue). (N = 180 cases, 340 controls).

The ROC curve showed DLK1 levels to be less predictive of SGA (Area under the receiver operating characteristic curve – AUROC = 0.578) than the ultrasound alone model (AUROC = 0.658) (**Figure 2**). Combining DLK1 levels with the prenatal ultrasound detection of SGA significantly increased the prediction of SGA than DLK1 alone (AUROC for ultrasound associated with maternal DLK1 levels = 0.675, p = 0.0002). However, DLK1 levels do not add much to the ultrasound model alone.

## Discussion

We showed the maternal level of DLK1 to be significantly lower during the second trimester of pregnancy for women who delivered a SGA newborn than that of women who delivered an AGA newborn. However, DLK1 levels were less predictive of SGA than ultrasound alone and combining DLK1 levels with ultrasound did not add much to the predictive value of ultrasound data alone. We observed a decrease in the level of DLK1 of the same magnitude in cases of SGA with placental vascular dysfunction as those due to other causes.

### DLK1 Levels in 2nd Trimester of Gestation Are Associated With SGA

Our results show an association between DLK1 levels in pregnancy during the 2^nd^ trimester of gestation and SGA, which was first described by Cleaton et al. for DLK1 levels measured during the 3^rd^ trimester of gestation (20). They reported lower levels of DLK1 at 36 WG for women who gave birth to SGA children in a study that included 45 women who delivered a SGA newborn and 43 matched controls. Recently, McDonald et al. confirmed lower DLK1 levels at 36 WG for women with SGA children in a cohort of 105 SGA and 245 controls. They also reported that DLK1 levels were already significantly reduced at 28 WG in women who delivered a SGA infant in a sub-group of 84 SGA cases and 132 controls (24). With a larger cohort and earlier in pregnancy than Cleaton and McDonald, our study confirms the association of DLK1 levels with SGA. The demonstration of such an association at an early stage in pregnancy should be beneficial for the management of newborns.

However, it is not well established whether the reduction of circulating maternal DLK1 levels during pregnancy causes fetal growth restriction or whether the low weight of the fetus and the placenta diminishes maternal DLK1 levels. An important clue to address this question would be provided by determining the source of maternal DLK1 during pregnancy. Circulating DLK1 levels are very low in non-pregnant women and substantially increase during pregnancy (20, 24, 28). Maternal DLK1 levels have been reported to be significantly higher in twin pregnancies than normal singleton pregnancies after 17 WG (29). Overall, these data suggest that maternal DLK1 measured during the 2nd and 3rd trimester originate from the fetus or the placenta. Cleaton et al. hypothesized that the source of maternal circulating DLK1 is the fetus, and not the placenta, based on data from murine models with conditional deletion of *Dlk1* (20). However, more recent data from humans have shown that DLK1 is present in the extra-embryonic tissue as early as 5 WG and remains detectable until birth (18, 30). Other studies have reported that placental expression of DLK1 is associated with fetal growth. One study reported a two-fold increase in the placental expression of DLK1 in large for gestational age newborns (birth weight > 90th percentile) (31) and another reported significantly lower DLK1 expression in the placentas of mothers who gave birth to SGA than those who gave birth to AGA infants (32). MacDonald et al. showed a positive correlation between maternal circulating DLK1 levels and placental weight and that DLK1 is secreted by the human placenta in *in-vitro* studies (24). Overall, these data at least partially support a placental origin of maternal circulating DLK1 in humans.

### Circulating DLK1 Levels at 26 WG Are Associated With SGA Independently of Signs of Placental Vascular Dysfunction

Data in the literature are contradictory on the association between maternal circulating DLK1 levels and placental vascular dysfunction. Cleaton et al. reported highly reduced DLK1 levels only in cases of SGA with placental vascular dysfunction, but no significant difference in maternal circulating DLK1 levels between SGA due to other causes and controls. MacDonald et al. found no relationship between circulating DLK1 levels and ultrasound measures of placental vascular dysfunction in a cohort of 63 women at 36 WG. Unlike Cleaton et al., we did not observe a significant difference in DLK1 levels between SGA with placental vascular dysfunction and SGA due to other causes in our cohort, in which 32.6% of SGA cases showed signs of placental vascular dysfunction. But we confirmed the results of MacDonald et al. in a larger cohort. However, the definition of SGA with placental vascular dysfunction is not consensual and differs widely depending on the study. In the study of Cleaton et al., SGA with placental vascular dysfunction was defined as SGA with high-resistance uterine artery flow, high-resistance umbilical artery flow, or low abdominal circumference growth velocity. Fifty-three percent of SGA cases were defined as SGA with placental vascular dysfunction in the study of Cleaton et al. In contrast, McDonald et al. reported only 6.3% of SGA with placental vascular dysfunction, defined as a low cerebroplacental ratio.

Regardless of birth weight, the association between DLK1 levels and placental vascular dysfunction has been documented by several studies. In a study that included 102 pregnant women, the median DLK1 serum concentration during pregnancy was significantly lower for women with preeclampsia than healthy pregnant women, even after adjustment for gestational age at blood sampling and birth weight (30). Schrey et al. reported lower placental expression of DLK1, primarily in the endothelium and cytotrophoblast, following immunohistochemistry of 40 placental biopsies of healthy pregnant women and speculated that aberrant cytotrophoblast development and endothelial dysfunction in preeclampsia may contribute to reduced placental expression of DLK1 in SGA fetuses.

### DLK1 as a Predictive Biomarker of SGA?

Using the combination of ultrasound data with a blood test that indicates placental dysfunction is a promising approach for the prediction of SGA (1). The measurements in maternal blood of various proteins or hormones produced by the placenta which are modified in the case of placental vascular dysfunction (pregnancy-associated plasma protein A - PAPP-A, soluble fms-like tyrosine kinase-1 -, sFLT1, placental growth factor - PlGF …) have been tested to identify women at risk of this adverse pregnancy outcome leading to FGR, stillbirth, and preeclampsia. But to date, none is used in clinical practice to predict the risk of SGA with or without FGR (1).

In our study, DLK1 level cannot be considered as a biomarker of SGA, as previously reported by Cleaton et al. and McDonald et al. (20, 24). DLK1 level is less predictive of SGA than ultrasound alone, and DLK1 level does not add much to the predictive value of ultrasound model alone.

The accuracy of detecting SGA for each woman could be enhanced by using ultrasound and following individual trends in the level of DLK1 during pregnancy. Indeed, we have observed a perfect exponential increase in the level of DLK1 when measured each four weeks during pregnancy in a woman who gave birth to an AGA child (unpublished data), offering promising perspectives.

### Strengths and Limitations

Our study is based on a well-characterized prospective cohort, which allowed us to measure maternal circulating DLK1 levels in a large sample of 193 cases of SGA and 370 matched controls. Maternal levels of DLK1 remained lower in women who delivered SGA than controls when considering the two centers of inclusion separately, showing that our results are robust. We found no association between maternal DLK1 level and placental vascular dysfunction for SGA children. This may have been due to the limited number of subjects with placental vascular dysfunction and the resulting lack of power. Nonetheless, the DLK1 levels for these subjects were very close to those of SGA due to other causes, making this hypothesis unlikely. Also, due to the design of the EDEN cohort and the information collected, data about the placental histopathology were not available.

## Conclusion

DLK1 levels at 26 WG are associated with SGA at birth, independently of signs of placental vascular dysfunction. However, DLK1 alone cannot predict the risk of SGA.

## Data Availability Statement

The data that support the findings of this study are available upon request from the EDEN steering committee. Readers may contact etude.eden@inserm.fr to request the data.

## Ethics Statement

Written informed consent was obtained twice from parents, once at enrolment and once after the child’s birth. The study was approved by the ethics research committee (Comité Consultatif de Protection des Personnes dans la Recherche Biomédicale, CCPPRB, N°02-70) of the Bicêtre Hospital and by the Data Protection Authority (Commission Nationale de l’Informatique et des Libertés).

## Author Contributions

AP, DM, AF, LP, YLB, BH, and IN contributed to the conception and design of this study. AP, EG, DM, AF, LP, YLB, FB, M-LS, BH and IN contributed to acquisition of the data or its analysis and interpretation. AP, DM, AF, LP, YLB, FB, M-LS, BH and IN contributed to drafting the article or critically revising it for important intellectual content. All authors contributed to the article and approved the submitted version.

## Funding

The EDEN study was supported by Foundation for Medical Research (FRM), National Agency for Research (ANR), National Institute for Research in Public Health (IRESP: TGIR cohorte santé 2008 program), French Ministry of Health (DGS), French Ministry of Research, INSERM Bone and Joint Diseases National Research (PRO-A), and Human Nutrition National Research Programs, Paris-Sud University, Nestlé, the French National Institute for Population Health Surveillance (InVS), the French National Institute for Health Education (INPES), European Union FP7 programs (FP7/2007–2013, HELIX, ESCAPE, ENRIECO, Medall projects), the Diabetes National Research Program [through a collaboration with the French Association of Diabetic Patients (AFD)], the French Agency for Environmental Health Safety (now ANSES), the complementary health insurance Mutuelle Générale de l’Education Nationale (MGEN), the French national agency for food security, and the French-speaking association for the study of diabetes and metabolism (ALFEDIAM). This work was supported by a grant (Grant for Growth Innovation, GGI) from Merck KGaA, Darmstadt, Germany. The authors declare that Merck KGaA was not involved in the study design, collection, analysis, interpretation of data, the writing of this article or the decision to submit it for publication.

## Conflict of Interest

The authors declare that the research was conducted in the absence of any commercial or financial relationships that could be construed as a potential conflict of interest.

## Publisher’s Note

All claims expressed in this article are solely those of the authors and do not necessarily represent those of their affiliated organizations, or those of the publisher, the editors and the reviewers. Any product that may be evaluated in this article, or claim that may be made by its manufacturer, is not guaranteed or endorsed by the publisher.
